# The “g” in Faking: Doublethink the Validity of Personality Self-Report Measures for Applicant Selection

**DOI:** 10.3389/fpsyg.2018.02153

**Published:** 2018-11-13

**Authors:** Mattis Geiger, Sally Olderbak, Ramona Sauter, Oliver Wilhelm

**Affiliations:** Department of Individual Differences and Psychological Assessment, Institute of Psychology and Education, Ulm University, Ulm, Germany

**Keywords:** faking, personality assessment, profile similarity metrics, cognitive abilities, applicant selection

## Abstract

The meta-analytic finding that faking does not affect the criterion validity of self-report measures in applicant selection suggests cognitive abilities are crucial to fake personality to an expected optimal profile in self-report measures. Previous studies in this field typically focus on how the *extent* of faking changes self-report measurement. However, the effect of faking *ability* is rarely considered. In Study 1 (*n* = 151), we link two questionnaires, the WSQ and the NEO-PI-R, to use them for later faking ability tasks. With O^∗^NET expert ratings and the linked questionnaires, we establish veridical responses of optimal personality profiles for both questionnaires. Based on this, in Study 2, we develop six faking ability task employing both questionnaires and three common jobs to fake for. To score the tasks, we introduce profile similarity metrics that compare faked response vectors to optimal profile vectors. The faking ability tasks were administered to a community sample (*n* = 210) who additionally completed measures of cognitive abilities, namely general mental ability, crystallized intelligence, and interpersonal abilities. For all, based on previous research, it can be argued that they should predict individual differences in faking ability. We establish a measurement model of faking ability and its relation to the other cognitive abilities. Using structural equations modeling, we find the strongest effect for crystallized intelligence and weaker effects for general mental ability and interpersonal abilities, all positively predicting faking ability. We show for the first time that we can measure faking ability with psychometrically sound techniques, establish a confirmatory factor model of faking ability and that it is largely explained by other cognitive abilities. We conclude that research supporting a positive link between self-reported personality and job performance is presumably confounded by cognitive abilities, because they are predictive of both faking self-reported personality and job performance. We recommend researchers to broaden their measurements with assessments of faking ability or other cognitive abilities (besides general mental ability) in research regarding applicant selection.

*“War is peace. Freedom is slavery. Ignorance is strength.” – George* Orwell (1949)

*Nineteen Eighty-Four – and self-report is truth. Doublethink*^1^ *the validity of self-reports.*

## Introduction

Numerous studies have demonstrated the predictive power of personality measures on job-related outcomes (e.g., Tett et al., 1991; Salgado, 1997; Hurtz and Donovan, 2000; Barrick et al., 2001). It is widely agreed that conscientiousness and neuroticism are predictive of job performance and that for specific jobs, specific Big-Five personality factors are important (Salgado, 1997; Hurtz and Donovan, 2000; Barrick et al., 2001).

In applicant selection, personality is typically assessed using self-report measures of typical behavior (e.g., Piotrowski and Armstrong, 2006). For the scope of this paper, when referring to the term personality, we refer to personality as typical behavior (c.f. Cronbach, 1949), assessed with self-report questionnaires, and categorized in the Big Five model. Based on lexical research, the Big Five subsume individual differences in personality under five traits – neuroticism, extraversion, openness, agreeableness, and conscientiousness (Goldberg, 1993). The reason why mostly self-report questionnaires are administered is probably because they are generally easy to administer and financially cheap relative to their assessment duration.

However, self-report questionnaires are vulnerable to various response biases and distortions (Ziegler, 2015). They can be categorized into unintended, unconscious, and intentional biases. Unintended biases, such as the error of central tendency (i.e., using only the middle anchors of a scale), are common, but they can be described and explained to participants and thereby reduced. Unconscious biases, such as biased responses due to item order, can be prevented through item randomization. However, intentional biases, such as faking, which are arguably a more frequent distortion in high stakes settings, cannot be easily controlled for entirely, either statistically or in a preventative sense. Following Sackett (2011), we define *faking* as a conscious and purposeful change in a person’s behavior, such as modifying responses on a self-report questionnaire so they are different from one’s “true” personality (Ziegler et al., 2011) to achieve a certain goal in a specific situation. In other words, faking means ignoring the usual instruction to answer honestly about one’s own personality.

With the influx of self-report personality questionnaires in applicant selection, research regarding how much faking occurs and how much faking influences personnel selection is crucial. The most obvious effect of faking is on mean personality levels. When instructed to, participants can easily fake their personality on a questionnaire (Furnham, 1990; Mersman and Shultz, 1998; Viswesvaran and Ones, 1999; Martin et al., 2002). In experimental settings, participants fake all Big-Five Personality factors to a similar amount (Viswesvaran and Ones, 1999). In non-experimental settings, such as among job applicants, faking is still highly prevalent, but it is subtler and more differentiated in general (Birkeland et al., 2006) and also differentiated depending on the job (Furnham, 1990). The Big-Five factors that most strongly predict job performance, namely conscientiousness and neuroticism, are faked the most with medium effect sizes (Birkeland et al., 2006). Therefore, it can be concluded that overall, self-report personality assessments in high stakes situations do not purely assess honest personality. On a side note, we want to stress that under experimental conditions, even if instructed to respond honestly, participants might not answer so. However, we assume that when reported under low stakes and assured anonymity, most reports should be honest, if instructed so. Thus, we will refer to personality scores acquired under “report honestly” conditions as “honest personality.”

There has been a long debate over the applicability of self-report personality questionnaires in applicant selection, which is still not resolved. Advocates of including personality questionnaires identify meta-analytic evidence illustrating the incremental predictive validity of personality questionnaires over intelligence tests on job-related outcomes, such as work performance (Schmidt and Hunter, 1998). However, opponents of personality questionnaires argue that faking distorts the selection decision because it can change applicant rank orders in the selection decision (Rosse et al., 1998) and the construct validity of the questionnaires (Schmit and Ryan, 1993). The latter raises the question of whether faked personality questionnaires are even measuring honest personality at all or something entirely different. Although studies showed that when disentangling disentangling faking and trait variance that trait variance is still predictive of real-life outcomes (Ziegler and Bühner, 2009; Ziegler et al., 2015), these studies only focused on faking *extent*. With faking *extent*, we refer to how *much* of the response is actually faked. We define this in contrast to faking *ability*, which refers to how *good or successful* somebody faked their responses. To handle and ultimately minimize these effects, it is necessary to better understand all aspects of faking, including all factors facilitating it. Next, we introduce these factors of faking.

Albeit no theoretical model of faking is considered conclusive, we consider Tett and Simonet (2011) performance model of faking the most advanced. It is a parsimonious model, but it covers all central aspects of faking. Please note that there are other models of faking (e.g., Ziegler, 2011), that include other traits predicting faking, such as employability (Ellingson, 2011). Although they certainly apply to real applicant selection situations, they might not always apply to research on faking. For example, employability presumably does not play a role when participants only pretend to apply for a job. Since Tett and Simonet’s model is parsimonious and generalizable on all faking research, we deem it the appropriate model for our studies.

Tett and Simonet argue faking is a unique type of performance: the intentional changing of one’s typical responses to achieve an intended goal. This intended goal, such as to get hired (Ziegler et al., 2011), can be used to evaluate the success of the faking performance. Based on an earlier performance model (Blumberg and Pringle, 1982), faking performance is viewed as the product of opportunity, motivation, and ability: Performance = Opportunity × Motivation × Ability. Meta-analytic evidence, introduced earlier, clearly shows that personality questionnaires are strongly faked, demonstrating that their response format offers a high opportunity. Furthermore, there is, as of today, no fail-safe method for detecting faking in questionnaires (Ziegler et al., 2011). Thus, it can be assumed that self-report questionnaires offer maximum opportunity to fake. At the same time, application sessions can be considered high stakes, so motivation can be considered at a maximum, too. Therefore, assuming opportunity and motivation are maximized and, thus, constant values, we expect ability to be the central influencing factor of faking performance on a self-report questionnaire in any applicant selection process.

Numerous studies found there is great variability in the *extent* to which individuals fake (e.g., McFarland and Ryan, 2000; Mueller-Hanson et al., 2006; Levashina et al., 2009). However, explanations for this variability based on faking *ability* are still rare. Engaging in faking behavior is cognitively demanding. Participants must identify those items where a high rating will present them in a favorable light for their desired job. This requires knowledge about the job (Vasilopoulos et al., 2000; Tett and Simonet, 2011) or knowledge about what personnel managers want to hear, referred to as the Ability to Identify Criteria (ATIC; König et al., 2006; Melchers et al., 2009; Kleinmann et al., 2011). MacCann (2013) found that faked personality scores correlated with crystallized and fluid intelligence more strongly than with honest personality scores, with correlations strongest with crystallized intelligence. Following recent findings on the dimensionality of knowledge (Schipolowski et al., 2014a,b), who repeatedly find a general factor of knowledge over various domains of knowledge, we hypothesize that the types of knowledge needed to fake personality are part of crystallized intelligence. Consequently, we expect crystallized intelligence to be a predictor of faking ability.

At the same time, applicants may worry about appearing credible toward the personnel manager and also that faking could be detected (Kuncel et al., 2011). To appear credible and specifically to understand and react to practitioners’ methods of detecting faking, participants need deductive reasoning, the core of general mental ability. Consequently, we hypothesize general mental ability to be a predictor of faking ability.

Finally, we consider faking ability to be an interpersonal ability. As mentioned above, faking is a form of deception, which is a key element in successful social interaction and itself clearly an interpersonal ability (Vrij, 2002). Similarly, ATIC, which can be classified as faking ability in job interviews, is also considered an interpersonal ability (König et al., 2006; Melchers et al., 2009; Kleinmann et al., 2011). Consequently, we hypothesize emotion perception to be a predictor of faking ability. In this research, we use emotion perception as a proxy of interpersonal abilities, because it is considered by some as the core of interpersonal abilities (Joseph and Newman, 2010; Hildebrandt et al., 2015). In sum, it can be argued that faking ability is related to crystallized intelligence, fluid intelligence, and interpersonal abilities. The strength of these relations, however, is so far mostly unknown.

The idea of considering individual differences in faking is not entirely new. However, only few studies have conceptualized faking as an *ability* and examined its relation to other cognitive abilities (Mersman and Shultz, 1998; Pauls and Crost, 2005; Raymark and Tafero, 2009). All these studies found positive relations of faking ability with general mental ability. Although faking was conceptualized as an *ability* in these studies, their operationalization is not without critique. A central aspect of ability measures is that their items have veridical answers and the score is calculated by comparing participants’ answers to the veridical answers (Cronbach, 1949). Mersman and Shultz (1998) and Raymark and Tafero (2009), however, assessed individual differences in the discrepancy between answers in honest and faking conditions. This approach has been criticized to be inadequate as a measure of *ability* (Pauls and Crost, 2005). We agree with this criticism and would classify this as an operationalization of faking *extent*. Furthermore, these studies had only a single faking ability task.

Pauls and Crost (2005), instead, used multiple faking tasks and faking scores adjusted for the honest scores. Specifically, they developed four faking tasks (general faking bad, general faking good, faking good applying as an organizational psychologist, and faking good applying as a clinical psychologist). As faking score they took the faked responses of the tasks, partialled out shared variance with honest personality and used the residual score for later analyses. We agree with their suggestion to use several tasks, but argue that the proposed scoring approach for faking *ability* should be improved. Considering the general rule of veridicality for ability tests, comparing a faked profile to an honest profile is not providing a comparison to a veridical answer, but instead to a typical answer. This, however, is the case if shared variance of honest scores is partialled out. Thus, this approach, too, measures faking *extent* and is not a measure of faking *ability*.

Identifying veridical answers for faking ability tasks is difficult. In tasks that simply instruct participants to fake good (or bad) for any job, there is no veridical response, because there is no optimal faking response valid for all existing jobs. Instead, as jobs require different personality profiles, optimal faking responses should vary depending on the job. Thus, to establish veridical answers to a faking task, one first needs to choose a particular job. For example, Pauls and Crost (2005) instructed participants to pretend they were applying for a job as a clinical or organizational psychologist. Next, the veridical answer for the task must be defined. This veridical answer is the perfect or optimal personality profile for this particular job. Such profiles are disputable. However, an approximation to veridicality might be achieved with expert ratings. Pauls and Crost (2005) used three experts to establish optimal personality profiles, although more experts would be desirable. These experts were asked to rate every item in a personality questionnaire with regards to which answer is representing the optimal personality trait level for their job. Pauls and Crost (2005) used the average rating of all experts per item as optimal personality profiles for a job. This results in profiles with some traits that require high levels, while others require medium or low levels. For different jobs, there are different patterns. If now a faked response on a personality questionnaire is compared to the optimal personality profile provided by experts, we would consider task and scoring adequate to measure faking *ability*. Again, this is in contrast to comparing faked responses to honest responses, which measures faking *extent*.

### Aims of the Current Studies

This paper aims to (a) develop sound measures of faking ability, (b) establish sound scoring methods for faking *ability*, (c) establish a measurement model of faking ability, and (d) explain individual differences in faking ability by established cognitive abilities. In Study 1, we choose a personality questionnaire with available expert ratings for numerous jobs and use them as optimal personality profiles for veridical responses. By linking this questionnaire to a second questionnaire and thereby generating optimal profiles for the second questionnaire, we broaden our item pool for faking ability tasks. To link the questionnaires, we ask a community sample to honestly respond to both questionnaires. Based on that data, we generate veridical answers for multiple faking ability tasks. In Study 2, we administer six faking ability tasks, based on both questionnaires and three distinct, commonly known jobs. We test these jobs along with measures of cognitive abilities in a second community sample and test competing scorings methods, establish measurement models of faking ability, and explain individual differences in faking ability with other cognitive abilities.

## Study 1

### Aims

The central aim of Study 1 was to contribute to study aims (a) and (b), specifically the development and scoring of multiple faking tasks. The latter aim is probably the more complex task, because numerous expert ratings are typically effortful to acquire. We use the average of multiple expert job incumbent ratings from the Occupational Information Network National Center for O^∗^NET Development, n.d.) database^2^, an enormous database of information (e.g., job requirements and characteristics) on a broad range of occupations. These expert ratings are given on a specific personality questionnaire, the Work Style Questionnaire (WSQ; Borman et al., 1999).

To develop multiple faking tasks, we identified two aspects of faking tasks that we can systematically vary. First, similarly as Pauls and Crost (2005), we created multiple tasks asking participants to fake for three different jobs. Second, there is a wide range of personality tests that can be used to design faking tasks. As a first test, we chose the WSQ, because O^∗^NET provides expert ratings directly for this questionnaire, which gives direct access to optimal personality profiles. As a second test, we chose the most frequently used personality test, the NEO-PI-R (Costa and McCrae, 2008). As there are no job incumbent ratings available for the NEO-PI-R such as O^∗^NET ratings for the WSQ, Study 1 strived to link both questionnaires. For this, we asked a general sample to honestly respond to both the WSQ and the NEO-PI-R. This linkage is used to select only the portion of NEO-PI-R items that best represent WSQ items. Finally, the linkage is also used to derive optimal profiles for these NEO-PI-R items.

### Materials and Methods

#### Sample

We recruited an online community sample (*n* = 232) through Amazon’s Mechanical Turk of persons residing in the United States (as indicated by their IP address) at the time of data collection. These samples are known to be more demographically diverse, relative to the more common student samples, with the reliability and validity of administered measures comparable to other types of samples (Buhrmester et al., 2011; Casler et al., 2013). Participants with any missing data were excluded (*n* = 81) because this could only occur if they aborted the study, which equaled a withdrawal of consent to the study terms. The final sample consisted of *n* = 151 participants (52% female) who were on average 39.5 (*SD* = 12.7) years old, mostly White non-Hispanic (90%, 5% Black non-Hispanic, 3% Asian, and 2% other). All listed English as their primary language, and the sample was diverse in educational attainment (12% High School or GED equivalent, 37% Some College, and 51% Bachelor or more than Bachelor’s degree).

#### Measures

All participants responded to tasks and items in the same order: WSQ, NEO-PI-R, demographic questions. There was no time limit.

##### Work Style Questionnaire (WSQ)

This 16-item self-report measure was developed for O^∗^NET and is intended to measure 16 work styles, each of which represents personality traits that are relevant for occupational performance. Items of the WSQ consist of the work style name (*Achievement/Effort*), a definition of this work style (*Establishing and maintaining personally challenging achievement goals and exerting effort toward mastering tasks*), and an instruction that varies depending on who receives the measure (e.g., experienced workers versus job seekers; Borman et al., 1999). For this study, instructions were asking the participant to describe themselves in personality task style fashion. That is, “*Please rate how much this characteristic describes you.*” Participants used a five-point Likert scale ranging from “*Not at all like me*” to “*Extremely like me.*”

##### NEO Personality Inventory Revised (NEO-PI-R)

The NEO-PI-R is an established and broad measure of typical behavior personality and consists of short first-person statements (e.g., *Once I start a project, I almost always finish it*). The task measures the Big-Five factors of personality with 30 underlying facets. Items are rated on a five-point Likert scale ranging from “*Strongly disagree*” to “*Strongly agree*” (Costa and McCrae, 2008).

### Results

#### Developing WSQ Subscales With NEO-PI-R Items

To obtain a selection of NEO-PI-R items for the faking NEO-PI-R tasks we correlated each WSQ item with each NEO-PI-R item and selected the eight NEO-PI-R items with the highest correlation to each WSQ item. We chose eight items to have scales the same length as the original NEO-PI-R facets, to have a sufficiently broad coverage of that particular work style, and to have enough items to facilitate later latent variable modeling. If less than eight items had a moderate correlation (*r* = 0.3) or higher with a WSQ item, no subscale was created for that work style (four work styles). With this effect size and an *n* of 151, with an α of 0.05, we had very high power (1-β) at 97%. The selected NEO-PI-R item codes per WSQ subscale are listed in a Supplementary File (Study 1_WSQ subscales.xlsx) on Open Science Framework (OSF^3^). It must be noted that some NEO-PI-R items appear in two WSQ subscales. Yet, given the fact that work styles, just as personality facets, are not orthogonal, this was expected and can be considered appropriate.

To assess the reliability and validity of the new subscales, we modeled a general factor for each set of NEO-PI-R items through confirmatory factor analysis and correlated that factor with the associated WSQ item. All models had acceptable model fit (for fit thresholds see the section “Statistical Analysis” in Study 2) and correlations were strong in effect size (Table 1). Additionally, each set of items had acceptable reliability, as indicated by ω > 0.70 (McDonald, 1999).

**Table 1:** Correlations between the relevant WSQ item and a single-factor measurement model of the relevant faking NEO subscale, factor reliabilities, and dominating NEO-PI-R factors in each faking NEO subscale.

Work style item/subscale	McDonald’s ω	*r* [95% CI]	Big-five factors
Achievement/effort	0.84	0.61 [0.47; 0.72]	C
Persistence	0.75	0.59 [0.48; 0.69]	C
Initiative	0.71	0.60 [0.50; 0.69]	C, E
Leadership	0.80	0.68 [0.59; 0.77]	E
Cooperation	0.83	0.71 [0.62; 0.79]	E, A
Concern for others	0.78	0.71 [0.62; 0.79]	A, O, E
Social orientation	0.86	0.66 [0.52; 0.77]	E
Self-control (R)	0.83	0.67 [0.57; 0.75]	N
Stress tolerance (R)	0.87	0.63 [0.52; 0.73]	N
Adaptability/flexibility	–	–	–
Dependability	0.76	0.58 [0.48; 0.68]	C, A
Attention to detail	–	–	–
Integrity	0.77	0.58 [0.47; 0.69]	C, A
Independence	–	–	–
Innovation	0.83	0.55 [0.41; 0.66]	O
Analytical thinking	–	–	–


*(R) indicates that the item was reversed to calculate the correlation. Empty cells with dashes are presented when no work style subscale could be formed for this WSQ item. In the *r* column, correlations of a WSQ item with the corresponding factor of its subscale are listed. Big-five factors, subscales consist of items taken from the listed factors; C, conscientiousness; N, neuroticism; A, agreeableness; O, openness to experience; E, extraversion. Order of factors indicates rank of number of items taken from this factor, with higher rank indicating more items taken from this factor. Bootstrapped confidence intervals (CIs) for correlations are reported in square brackets.*

#### Deriving NEO-PI-R Optimum Profiles

Next, to derive optimal profiles for the NEO-PI-R items, they were regressed on the corresponding WSQ item (using linear regression), and the intercept (a) and linear slope (b) scores were retained. The generalized regression equation was NEO_i_ = *a_i_* + *b_i_* ^∗^ WSQ_i_. To calculate the optimal profile scores for each NEO-PI-R item, we used those parameters and replaced WSQ_i_ in the regression equation with the respective average WSQ expert ratings from the O^∗^NET database. The excel file used to calculate this (Study 1_optimum profile item level faking NEO.xlsx) is available from our OSF repository^3^. We will henceforth refer to these O^∗^NET expert job incumbent ratings and their NEO-PI-R derivatives as optimal profiles. Please note that this method allows deriving an optimum score that is outside the bounds of the response scale, e.g., >5. Although this did not happen (overall Max_OP_ = 4.40 and Min_OP_ = 1.58), we would recommend adjusting the value to the minimum or maximum value of the response scale, if this occurs.

### Conclusion

In this study, we successfully linked the WSQ and the NEO-PI-R. We developed eight-item NEO-PI-R scales for 12 work styles, which showed strong relations with the corresponding work style item. This was done to contribute to our aims (a) and (b), specifically the development of sound faking measures and corresponding sound scoring methods. The linkage of both questionnaires provides us with items for WSQ- and NEO-PI-R-based faking tasks. With O^∗^NET expert ratings, we have optimal profiles available for WSQ-based faking tasks. Through the linkage, we also generated optimal profiles for NEO-PI-R items. In the following study, we use the outcomes of Study 1 to develop six faking ability tasks.

## Study 2

### Aims

The central aims of this study were to compare competing scoring procedures (aim b), establish measurement models of faking (aim c), and use them to test predictors of individual differences in faking ability (aim d). By selecting three jobs (pilot, television/radio announcer, and tour guide) per task (faking ability task based on WSQ and NEO-PI-R) we develop and administer six faking personality tasks. We introduce profile similarity metrics (PSM) to score the faking tasks, which allows us to compare faked responses to the optimal profiles derived in Study 1. The competing scores are evaluated psychometrically and tested in confirmatory factor models. Additionally, we administer measures of fluid intelligence, crystallized intelligence, and emotion perception ability. Based on work introduced before, we hypothesize that crystallized intelligence, general mental ability, and emotion perception ability predict individual differences in faking ability.

### Materials and Methods

#### Sample

Participants were recruited through Amazon’s Mechanical Turk with the same sampling restrictions as Study 1. A total of 299 participants answered all questionnaires, which was mandatory to receive credit for participation. To reduce noise in the sample (Oppenheimer et al., 2009), we excluded the data from 89 participants who incorrectly answered one or more attention check items (e.g., “*What job are you pretending to apply for? 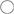 A superhero 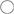 A tour guide*”). The exclusion rate of 30% is below the average exclusion rate of other studies introducing attention check items (Oppenheimer et al., 2009) and is usual for online community samples. The final sample consisted of 210 participants (78% female) who were on average 37.5 years of age (*SD* = 12.0), were mostly White non-Hispanic (83%, 6% Black non-Hispanic, 4% Asian, and 7% other), and varied in their educational attainment [Less than High School (1%), High School or GED equivalent (8%), Some College (34%), Bachelor or more than Bachelor’s degree (47%), 10% did not report their educational level]. Participants primarily listed English as their native language (99%). Since non-native speakers solved all attention checks correctly, we assume their English proficiency to be sufficient to understand all tasks and they were retained in the final sample.

#### Measures and Availability

All tasks were programmed as an online survey on Unipark^4^ and presented in an alternating order so that no same type of tasks (e.g., two tasks on faking ability based on the same personality questionnaire but for differing job profiles) were presented consecutively. All participants responded to all tasks. There was no time limit. Completion of the study took about 1 h. The order of measures was: Trait-Self Description Inventory-42i short form (TSDI-42i; Olaru et al., 2015), Job Knowledge Pilot, Faking WSQ Pilot, BEFKI-gf, Honest NEO, Faking NEO Pilot, Composite Emotions Emotion Perception Task, Faking NEO TV/radio announcer, Job Knowledge TV/radio announcer, BEFKI-gc, Faking WSQ TV/radio announcer, Faking WSQ Tour Guide, Honest WSQ, Visual Search Emotion Perception Task, Faking NEO Tour Guide, Job Knowledge Tour Guide, and Demographic Questionnaire. The instruments are explained in detail below.

Our data collection was solely based on *a priori* hypotheses that individual differences in faking ability exist and they can be explained by other cognitive abilities. Test materials for the cognitive measures (fluid and crystallized intelligence, emotion perception ability) are copyrighted but available from the respective authors for research purposes. Examples of instructions of the faking measures are uploaded to the open science framework^3^. Items of the faking tasks were taken from the questionnaires that had to be faked and are available from their respective sources.

##### Berliner Test for Fluid and Crystallized Intelligence (BEFKI) 8–10 figural fluid intelligence

This 16-item measure of fluid intelligence presents a series of figures and participants are asked to decide which two figures complete the series (Wilhelm et al., 2014b). For each of the two consecutive figures, subjects choose from three options in a multiple-choice setting, and participants must correctly select both consecutive figures in order to get the item correct. The BEFKI-gf has been shown to be very reliable with ω = 0.87.

##### BEFKI 8–10 crystallized intelligence

In this measure of crystallized intelligence, participants respond to 32 multiple-choice questions on general knowledge. It was translated to English for the present study, as well as adapted for the US culture and reduced to half of the original items. As we anticipated the sample to be broadly distributed in terms of intelligence, we chose the 8–10 version of both BEFKI tasks as they contain a sufficiently broad range of item difficulties (Wilhelm et al., 2014b). Since this translation differs substantially from the German original version and was translated for this study, we cannot report reliabilities from previous studies.

##### Visual search for faces with corresponding emotion expressions of different intensity

This measure of emotion perception ability asks participants to decide which target faces do not express the same emotion as the majority in a 3 × 3 grid of emotional faces of the same identity (Wilhelm et al., 2014a). The number of target faces is indicated for each trial. A short-form version included 20 trials, with four trials for each of the five basic emotions (anger, disgust, fear, sadness, and surprise); the trial emotion was determined by the emotion that was presented in the majority of faces. There were no trials for happiness because they have been shown to have ceiling effects (Wilhelm et al., 2014a). The full version of this task has an acceptable reliability of ω = 0.64.

### Faking Personality

To achieve an appropriately broad measurement of faking ability, several faking tasks were developed for this study. The aspects of personality that are faked differ depending on the job of interest (Furnham, 1990). We selected three commonly known jobs with distinct work style patterns: pilot (O^∗^NET code: 53-2012.00), tour guide (O^∗^NET code: 39-7011.00), and television/radio announcer (O^∗^NET code: 27-3011.00). These jobs vary substantially in the rank order of importance of work style; they also include work styles with only medium (e.g., 3) or even lower importance for the job. Thereby, they have complex patterns of work styles that will penalize participants who generally fake all work styles to a maximum in the faking tasks. All expert scores on all jobs in O^∗^NET are available from their website^5^. We must note that the chosen jobs are clearly not the only combination of distinct jobs and may not be the most distinct combination possible. Additionally, in our selection, at least 25 experts had to have rated that job and the job had to be commonly known.

In addition to varying jobs, we designed two types of faking tasks. The first, labeled “faking WSQ,” directly employs all WSQ items and their personality dimension-like labels. The second, labeled “faking NEO,” employs NEO-PI-R items linked to four work styles from Study 1 (Table 2). Within a faking NEO task, the work styles were chosen to vary in terms of O^∗^NET job incumbent ratings of relevance to increase item difficulty.

**Table 2:** Work styles (faking WSQ) or work style subscales (faking NEO) assessed for each job and each type of faking personality task.

	Pilot	Tour guide	TV/radio announcer
Faking WSQ	All work styles	All work styles	All work styles
Faking NEO	Self-control (4.7)	Self-control (4.5)	Integrity (4.4)
	Persistence (4.1)	Cooperation (4.5)	Stress tolerance (4.3)
	Concern for others (4.1)	Leadership (3.7)	Innovation (4.0)
	Innovation (3.5)	Achievement/effort (3.4)	Leadership (3.7)


*Values in brackets represent the mean expert scores that are desirable for the given job, taken from the O^∗^NET database.*

#### Faking WSQ

In the first type of task, participants were given all 16 WSQ items (Borman et al., 1999) and asked to select the response option that would support their idea of the ideal personality for one of the three chosen jobs. For reasons of standardization and scoring, we used a 50-point Likert response scale with a slider using the original WSQ anchors ranging from “*Not important*” to “*Extremely important.*” The 50-point Likert scale was necessary to calculate adequate PSM. Because the optimal profile scores are mean scores among expert ratings, they were not natural numbers (1, 2, 3, 4, 5), but rational numbers (e.g., 4.7, 3.4, etc.). Using standard five-point Likert scales would have required rounding the optimal profile scores, which would have resulted in a huge loss of information and a probably higher amount of measurement error in the PSM. For later analyses, the values from the 50-point Likert scales were divided by 10.

#### Faking NEO

In the second type of task, participants were given four work style subscales, each including the eight NEO-PI-R items identified in Study 1. Like with the faking WSQ tasks, participants were instructed to select the response option that would support their idea of the ideal personality for the job in question and we used a 50-point Likert response scale with the original NEO-PI-R anchors ranging from “*Strongly disagree*” to “*Strongly agree.*” In each faking NEO task, a distinct set of four work style subscales were chosen to be faked. The four subscales were chosen to vary substantially in terms of their job relevance, indicated by their mean expert rating of the related WSQ item. We only chose four subscales (i.e., 32 items) to keep a considerable length for every task. Furthermore, per job, we only selected subscales with no overlap in items to avoid local stochastic dependencies. The related WSQ item and the respective mean expert ratings are reported in Table 2. Complete faking tasks are available from the first author upon request.

In both task types, participants received a lengthy instruction explaining they should imagine they are applying for the described job, that this is their dream job, and that they should adjust their responses in a way that they deem appropriate to receive a job offer. After an additional short emphasis on the importance of this application, participants were asked to first answer two attention check items and then complete the faking WSQ task or faking NEO task (specific to that job) to maximize their chances of getting hired. An example of these instructions for each job and the attention check items are provided in the Supplementary Material uploaded to the Open Science Framework^3^.

#### Scoring Faking Ability

The veridical response of the faking WSQ tasks was the mean score of the expert job incumbent ratings from the O^∗^NET database, retrieved in March 2015. The veridical response for the faking NEO-PI-R tasks was developed in Study 1 (see the section ‘Deriving NEO-PI-R Optimum Profiles’ in ‘STUDY 1’).

Ability was scored using PSM (Legree et al., 2010, 2014). We use two scores: The first is *elevation*, the absolute difference between an average participant’s response across all relevant items and the average of the optimal profile. The second score is, *shape*, the correlation between the participant’s response and the optimal profile. Please note that there are competing methods for the computation of elevation and shape (c.f. Cattell, 1949; Cronbach and Gleser, 1953; Abdel-Aty, 1960; Cohen, 1969). However, we consider Legree et al. (2014) as the most advanced and therefore used their formulae. Namely, for elevation, we subtracted the mean of the response vector (of a task or subscale) from the mean of the corresponding optimal profile vector and used them as absolute values. Thus, low values indicate proximity to the optimal profile and therefore high faking ability. In shape (computed as Pearson’s *r*), interpretation is reversed, with negative scores indicating opposing patterns, zero scores indicating orthogonal patterns, and positive scores indicating similar patterns. Please note that if participants had a faked response vector with zero variance in a single task or within a subscale, shape could not be computed and their value was set to missing. This resulted in a total of 13 missing observations (1% of all shape data) in the final dataset.

**FIGURE 1:**
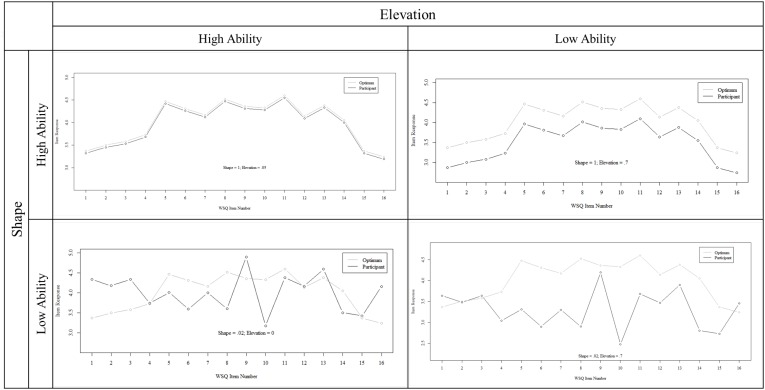
Four examples of combinations of high and low elevation and shape to illustrate the scores’ distinctiveness.

We want to acknowledge that although Legree et al. (2014) showed that elevation is contained in the equation of shape, we disagree that they are redundant. We estimated elevation separately because shape and elevation ability levels can differ substantially in one response profile. Four illustrations of high/low elevation and shape are exemplified in Figure 1. As visible, elevation represents the *proximity* of two profiles, or the ability to correctly estimate how much all personality factors are valued for a particular job. In contrast, shape represents similarity in profile *patterns* or the ability to correctly estimate which items are the most important and which are the least important. Thus, elevation requires absolute and detailed knowledge on levels in profiles, however wrong estimations in some items might be compensated by very precise estimation elsewhere. In shape, not the precision of every single guess, but the relation (differences) between the levels are important.

Due to the nature of absolute difference scores, the elevation scores typically showed right-skewed distributions. To achieve better distributional properties, which are necessary for the following analyses, we took the square root of the elevation scores.

#### Excluded Measures

We also administered a short version of the “Identification of Emotion Expressions from Composite Faces” task (Wilhelm et al., 2014a) and a newly developed job knowledge task for the specific jobs to be faked in the faking tasks. These two tasks had strong psychometric limitations (extreme ceiling effects and related severe modeling issues) and were therefore excluded from the analysis. Since both tasks had been shortened or developed uniquely for this study, these issues could not be solved in the present study and an exclusion of their data was decided as the most appropriate solution.

Furthermore, the 68 NEO-PI-R items that were used for the faking NEO task were also administered and participants were asked to respond honestly. This selection of items included 16 items assessing neuroticism, 13 extraversion, 9 openness, 14 agreeableness, and 16 conscientiousness. Here, the usual instruction of the NEO-PI-R was used (Costa and McCrae, 2008), but participants answered on a 50-point Likert scale with a slider ranging from “*Strongly Disagree*” to “*Strongly Agree.*” A second honest personality assessment was conducted using a shortened version of the TSDI (TSDI-42i, c.f. Olaru et al., 2015) with original instructions and response options. For a third honest personality assessment, the WSQ was also assessed under report honest condition. As this manuscript focuses on faking ability for which a comparison to an optimal personality profile is required, and not a participant’s honest personality profile, the honest personality measures are not further considered in the manuscript.

#### Statistical Analyses

All statistical analyses were conducted using R 3.2.3 (R Core Team, 2015). Differences between standardized orthogonal regression weights (interpreted as correlations) were estimated using the paired.r function in the *psych* package (Revelle, 2017). Latent analyses were conducted using the package *lavaan* (version 0.5-20; Rosseel, 2012), and McDonald’s (1999) estimate of factor saturation, ω, an indicator of reliability, was estimated using the package *semTools* (version 0.4-13; semTools Contributors, 2016). All latent models were estimated with maximum-likelihood (ML) estimation and missing values were handled with the ML estimation algorithm, which is implemented in lavaan. Factors were identified using the effects coding method, as described in Little et al. (2006). When interpreting model fit, we define CFI ≥ 0.90 and RMSEA < 0.08 as acceptable (Bentler, 1990; Steiger, 1990). We will refer to regression weights between latent variables as γ.

Since the typical significance tests of SEM parameters are dependent on how the model is identified, we used the likelihood ratio test introduced by Gonzalez and Griffin (2001) to estimate the statistical significance of relations between latent variables. Following limitations described by Stoel et al. (2006), we adjusted the degrees of freedom of the χ^2^-distributions of these tests accordingly. With *n* = 210, α = 0.05, and a moderate power (1-β) of 80%, we had enough sensitivity to detect bivariate manifest correlations as small as *r* = 0.19. For the χ^2^(0.5)-tests, with the same settings on *n*, α, and power, we could detect differences as little as χ^2^(0.5) = 3.841. Ninety-five percent confidence intervals were estimated via bootstrapping with 1000 draws each. As general significance threshold for this paper, we set α = 0.05. All power analysis was conducted using GPower (Faul et al., 2009). We provide the dataset and R syntax for profile similarity scoring and latent analyses in the Supplementary Material uploaded to the Open Science Framework^3^.

### Results

#### Descriptive Statistics of Faking Ability

Zero-order covariance and correlation matrices of all scored measures are reported in the Supplementary Material in the Open Science Framework. Descriptive statistics are summarized in Table 3. All faking scores were normally distributed and differed from one another in their mean levels, indicating differences in task difficulty. For shape, high values indicate high ability. For elevation, low scores indicate high ability. Thus, the faking WSQ tasks were easier overall than the faking NEO tasks. Faking applying as a pilot was the easiest job to fake.

**Table 3:** Descriptive statistics of both faking scores from all six faking tasks.

Profile similarity metric	Job	Faking task type	*M*	*SD*	Min	Max	Skew	Kurtosis
Elevation	Pilot	WSQ	0.58	0.25	0.07	1.17	0.22	-0.79
(square root transformed)		NEO	0.83	0.13	0.43	1.11	-0.51	-0.04
	Tour guide	WSQ	0.63	0.24	0.07	1.19	-0.28	-0.66
		NEO	0.92	0.17	0.38	1.17	-1.01	0.44
	TV/radio announcer	WSQ	0.61	0.24	0.08	1.14	-0.32	-0.68
		NEO	0.79	0.15	0.32	1.18	-0.29	0.12
Shape	Pilot	WSQ	0.59	0.18	0.04	0.89	-0.75	0.27
		NEO	0.26	0.16	-0.16	0.71	-0.40	0.43
	Tour guide	WSQ	0.39	0.30	-0.74	0.88	-1.19	1.73
		NEO	0.23	0.17	-0.24	0.62	-0.41	-0.32
	TV/radio announcer	WSQ	0.14	0.23	-0.51	0.66	-0.32	-0.07
		NEO	0.19	0.17	-0.37	0.59	-0.55	0.24


**M*, arithmetic mean; *SD*, standard deviation; Min, minimum; Max, maximum.*

#### Measurement Model of Faking Ability

**FIGURE 2:**
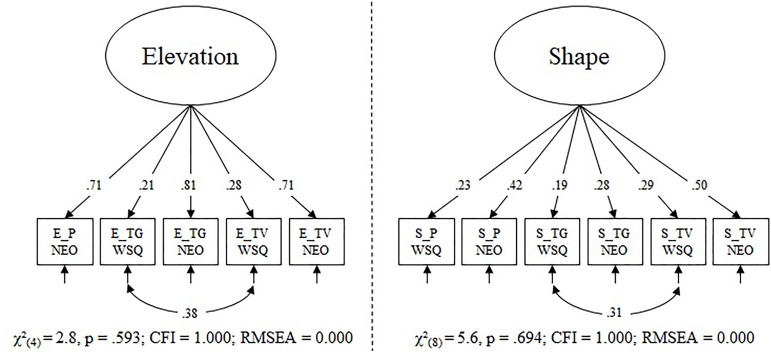
Final confirmatory factor models for elevation score of faking ability and shape score of faking ability. Loadings and correlations are fully standardized. The following abbreviations were used to generate the indicator codes: E, elevation; S, shape; P, pilot; TG, tour guide; TV, TV/radio announcer; WSQ, Work Style Questionnaire; NEO, NEO-PI-R. The dotted line indicates that the models were estimated separately.

Elevation and shape were modeled as separate latent variables, thereby assuming that a general faking ability each underlies individual differences in elevation or shape of faking ability. The raw model of elevation had poor fit [χ^2^(9) = 44, *p* < 0.001; CFI = 0.855; RMSEA = 0.136]. In a *post hoc* evaluation, the misfit could be explained by a negative loading on the faking WSQ – pilot indicator (λ_1_ = -0.194, *p* = 0.026) and modification indices showed a correlation between the error terms of faking WSQ – tour guide and faking WSQ – TV/radio announcer would result in a large improvement to the χ^2^. For the final elevation model, we therefore excluded the faking pilot WSQ indicator and added the before mentioned correlation. We believe the modeling issues are due to unknown problems with the faking WSQ pilot indicator and due to shared method variance for the other faking WSQ tasks. The final elevation model had a very good model fit (Figure 2). All loadings and the error term correlation were significant. The factor had acceptable factor saturation (ω = 0.54) and significant factor variance (*p* < 0.001).

The raw model of shape had an acceptable RMSEA = 0.050 and a non-significant χ^2^-test of model fit [χ^2^(9) = 16, *p* = 0.059], but a poor CFI = 0.824. We therefore allowed the equivalent error term correlation as in the elevation model (which also had the highest modification index in the present model), resulting in a model with very good fit to the data (Figure 2). All loadings and the error term correlation were significant. The factor had low factor saturation (ω = 0.33); however, the factor variance was significant (*p* = 0.003). In both models, the faking NEO tasks were stronger indicators of the factor than the faking WSQ tasks. In a last step, we modeled both latent variables in a single confirmatory factor analysis and correlated both factors. The correlation was not significant [*r* = 0.16, χ^2^(0.5) = 1.589, *p* = 0.104; CI (-0.222; 0.517)]; thus, we assume the two faking factors to be independent and modeled them separately in the final analyses.

#### Measurement Model of Intelligence

Fluid intelligence, crystallized intelligence, and emotion perception ability were also modeled as latent variables based on parceled indicators. For fluid intelligence, items were randomly assigned to one of three parcels, for which average performance was calculated (gf_1_, gf_2_, gf_3_). For crystallized intelligence, we estimated average performance for the three knowledge domains (gc_Sci_, gc_Hum_, gc_Soc_; for details see Wilhelm et al., 2014b), and for emotion perception ability we estimated average performance for the emotion congruent trials (ep_a_, ep_d_, ep_f_, ep_sa_, ep_su_). Following established intelligence models (McGrew, 2009) with recent advances in the study of emotional abilities (MacCann et al., 2014), we considered fluid intelligence, crystallized intelligence, and emotion perception ability to all be indicators of general intelligence.

**FIGURE 3:**
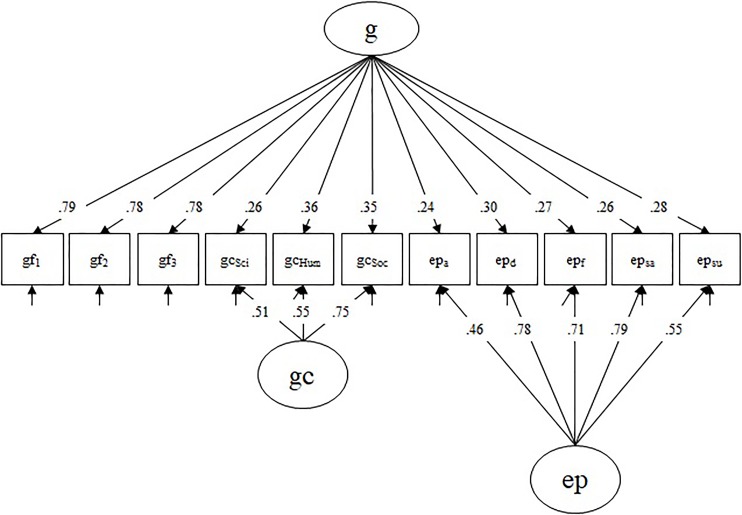
Confirmatory bifactor model of the cognitive covariates. Gf indices are taken as reference for the g factor. Loadings are fully standardized. All factors are orthogonal. g, general mental ability; gf, fluid intelligence; gc, crystallized intelligence; ep, emotion perception ability; Sci, science; Hum, humanities; Soc, social sciences; a, anger; d, disgust; f, fear; sa, sadness; su, surprise.

Performance on the cognitive tasks was modeled with a bifactor structure (Figure 3). We modeled a general factor of mental ability (g), indicated by all cognitive parcels. Due to the dominant role of fluid intelligence in intelligence theory, the gf-parcels served as the reference for the g-factor. This means, as is visible in Figure 3, that no nested factor for gf was modeled so gf is the driving factor in g and gf-specific variance is not contaminating the specific factors of gc and emotion perception. We also modeled a nested crystallized intelligence factor (gc), indicated by gc_Sci_, gc_Hum_, gc_Soc_, and a nested emotion perception ability factor (ep), indicated by the five emotion perception parcels. With this structure, these orthogonal nested factors represent variance specific to that ability only, excluding variance shared with the general mental ability g-factor. The model had an acceptable fit with χ^2^(36) = 66, *p* = 0.002; CFI = 0.964; RMSEA = 0.063. All loadings were statistically significant and the factors had decent factor saturation (ω_g_ = 0.83, ω_gc_ = 0.67, ω_ep_ = 0.83).

#### Disentangling Faking Ability

Next, we modeled two structural equation models (SEMs), each letting all cognitive factors of the bifactor model predict either elevation (SEM1) or shape (SEM2; Table 4). The regressions were estimated with latent variables within the SEM that contained the before mentioned (final) measurement models of both faking scores and the cognitive covariates. It should be noted that, due to the bifactor structure of cognitive abilities, all predictors were orthogonal.

**Table 4:** Results of the structural equation models (SEM1) and (SEM2) of cognitive abilities predicting elevation and shape in faking ability.

	General mental ability	Crystallized intelligence	Emotion perception ability
**(SEM1) elevation**			
γ (standardized) [95% CI]	0.077 [-0.102; 0.257]	-0.069 [-0.291; 0.133]	0.118 [-0.044; 0.272]
χ^2^	0.805	0.538	1.912
*p*	0.185	0.232	0.084

Model fit:	$\chi_{\left(92\right)}^{2}$ = 146, *p* < 0.001; CFI = 0.951; RMSEA = 0.053		
*R*^2^	0.024		

**(SEM2) shape**			
γ (standardized) [95% CI]	0.218 [-0.061; 0.524]	0.495 [0.036; 1.189]	0.316 [0.012; 0.666]
χ^2^	3.107	9.973	6.887
*p*	0.039	0.001	0.005

Model fit:	$\chi_{\left(107\right)}^{2}$ = 130, *p* = 0.060; CFI = 0.973; RMSEA = 0.032		
*R*^2^	0.393		


*γ represents standardized regression weights of latent constructs predicting the latent factor of faking. Bootstrapped CIs are reported in square brackets.*

The elevation equation (SEM1) revealed very low γs close to zero. Only γ_ep_ was greater than 0.1, but like the others, it was not significant. Results indicate that the measured cognitive abilities were unrelated to individual differences in elevation (SEM1; total *R*^2^ = 0.024; Table 4). However, general mental ability was weakly related with shape (γ_g_ = 0.218, *p* = 0.039), emotion perception ability was moderately related (γ_ep_ = 0.316, *p* = 0.005), and crystallized intelligence was strongly related (γ_gc_ = 0.495, *p* = 0.001; total *R*^2^ = 0.393). Overall, while cognitive abilities were unrelated to faking ability elevation, a substantial proportion of variance in faking ability shape could be explained by other cognitive abilities.

Because there were no significant relations for faking ability elevation, we compared effect sizes between SEM1 and SEM 2 and within SEM2. Since the effects were standardized regression weights of orthogonal predictors, we interpreted them as correlations and calculated the difference between paired correlations (using one-tailed *p*-values). Since shape and elevation were not significantly related, we considered them to be unrelated in the tests of difference of correlations. The effects of gc (*r*_diff_ = 0.564; *z* = 6.224, *p* < 0.001) and emotion perception ability (*r*_diff_ = 0.198; *z* = 2.123, *p* = 0.017) on shape were significantly stronger than for elevation. The effect of g, however, did not significantly differ between the two models (*r*_diff_ = 0.141; *z* = 1.469, *p* = 0.071). Within the SEM2, the correlation of shape with gc was significantly different than the correlation of shape with g (*r*_diff_ = 0.277; *z* = 3.267, *p* = 0.001) and emotion perception ability (*r*_diff_ = 0.179; *z* = 2.192, *p* = 0.014), but correlations of shape with emotion perception ability and g did not differ in magnitude (*r*_diff_ = 0.098; *z* = 1.075, *p* = 0.141).

## Discussion

Faking is a crucial confounding factor in any high-stakes assessment situation, such as in applicant selection. In frequently used self-report questionnaires, faking is a highly prevalent phenomenon (Birkeland et al., 2006) that distorts the construct validity of the measurement (Schmit and Ryan, 1993). Consequently, it is vital for practitioners and researchers alike to understand determinants of faking. Following Tett and Simonet (2011) model of faking, we identify faking ability as the crucial factor for faking. There are several previous studies exploring faking under the label faking ability. However, given our distinction between faking *extent* and faking *ability*, we argue that this research primarily addresses faking *extent*. Instead of comparing faked response vectors to honest response vectors (Mersman and Shultz, 1998; Raymark and Tafero, 2009), studying faking *ability* requires comparing faked response vectors to veridical response vectors (Cronbach, 1949). As veridical response vectors, we introduced optimal personality profiles based on expert ratings from O^∗^NET. Striving to further understand faking ability, we set four aims for this study: (a) establish sound measures, (b) establish sound scoring procedures, (c) establish a measurement model, and (d) test cognitive abilities as predictors of individual differences in faking ability.

To answer aim (a), we developed and evaluated six tasks that assess individual differences in faking ability, with veridical answers, and to answer aim (b) we applied PSM for scoring. For each combination of faking task and scoring method, we found clear individual differences and, with a square root transformation of the elevation score, adequate distribution properties. Answering aim (c), the establishment of a measurement model, we modeled individual differences in faking ability as two orthogonal latent variables, one for each PSM (elevation and shape), indicating that these scores each represent a distinct ability. The empirical orthogonality of the faking factors is not surprising. One explanation is their obvious difference in meaning. Shape represents the relative importance of a particular trait, or more specifically, the relative distance between the traits or single items to be faked. Elevation represents the exact level of difference between the profiles.

For the final aim (d), we tested cognitive abilities as predictors of faking ability. We hypothesized that crystallized intelligence, general mental ability, and interpersonal abilities will predict faking ability. Again, there are differences between the two faking ability scores. With structural equation modeling, we found that elevation cannot be explained by any of our predictors. However, a substantial portion of shape was explained by these cognitive abilities and effect size differences between shape and elevation were significant for crystallized intelligence and emotion perception ability. In shape, primarily crystallized intelligence, but also to some extent emotion perception ability and general mental ability, facilitate the ability to adjust one’s shape of a response pattern, which is in line with previous studies and theories presented above (Kleinmann et al., 2011; MacCann, 2013). While crystallized intelligence possibly facilitates an understanding of relative distance, as represented in shape, more specific and extremely high-level knowledge about a job might be necessary to facilitate elevation. Another explanation for the null-effects on elevation might be that individuals have different understandings of a response scale, or may compare themselves to different groups of people, with different mean levels on that attribute, which would only affect elevation, but not shape. So far, the cognitive abilities that explain elevation remain unclear and should be investigated in future research. Furthermore, we were successful in accounting for some, but not all, of the variance in faking ability. Obviously, additional predictors of faking ability tasks, besides the cognitive abilities studied here (e.g., employability or narcissism), might account for additional variance. Future studies might investigate the predictive power of these traits.

### Limitations

Some caveats must be considered when interpreting these results. One limitation is the rather low reliabilities of the faking factors. Presumably, this is mainly driven by high proportions of method variance related to the WSQ items. This is indicated by the error correlation between the other two WSQ items for both elevation and shape. One explanation of the high method-specific variance might be because in the WSQ task item instruction participants were asked to rate the importance of a trait for a job, which is different from the NEO-PI-R instruction that asks for agreement or disagreement to first person sentences. Another explanation might be the test length, with the much shorter WSQ. However, we still consider the tasks to be appropriate measures of faking ability because the instructions presented the tasks as maximal performance faking ability tasks. Thus, we assume that a good faker should respond in a comparable way, no matter if the item instruction aims at the importance of a trait or at describing oneself. Still, the reliability of the faking factors is unsatisfactory. Latent variable analysis is best suited to analyze such data and analysis based on manifest faking scores should be avoided. Future research should seek to further improve the measurement of faking ability. The WSQ-specific variance might be reduced by equating the item instructions to other tasks. Additionally, with different contexts, other questionnaires, “faking bad” tasks and new response formats, broader and more reliable assessments of faking ability could be achieved.

Another aspect probably influencing the validities in this study is the measurement of interpersonal abilities. Although an emotion perception ability task can clearly function as a proxy for interpersonal abilities, it is too narrow of a measure to cover the whole range of interpersonal abilities. In this study, measurement of interpersonal abilities was limited to only emotion perception ability due to a limited testing time and task presentation constraints in online studies. We recommend future studies to include more measures of interpersonal abilities and specifically more deception-related measures of interpersonal abilities, such as emotion expression or regulation ability.

Finally, we want to stress that a bigger sample, resulting in more statistical power of the presented tests, would have been desirable. Some tests, specifically for the correlation between the faking factors, the γs for the elevation model, and the γ_g_ were underpowered. Yet, with the given sample, by thoroughly eliminating unreliable responders from the sample by a series of attention check items, we sought to reduce error in the sample and thereby improve power to an acceptable level. Furthermore, the sample in Study 2 was not generalizable in terms of participant sex. Thus, we strongly encourage other researchers to replicate our effects with bigger and more general samples.

## Conclusion

In this paper, we show how to set up a study to measure faking *ability* rather than faking *extent*. To achieve this, we applied PSM and optimal profiles to appropriately score participants’ responses as an ability. We also demonstrate the major relevance of distinct cognitive components in faking ability. This has implications for future faking research.

It is well known that cognitive abilities predict job performance and that personality has incremental predictive power over cognitive abilities (Schmidt and Hunter, 1998). However, our findings challenge this view. Based on Tett and Simonet’s (2011) model, we argue that individual differences in faking are mostly driven by individual differences in faking ability, because in settings, such as personality questionnaires used in applicant selection, motivation, and opportunity to fake can be considered at a constant maximum. In Study 2, we find that faking ability can be measured appropriately and that it is largely explained by cognitive abilities. In sum, job performance and faking ability have a common predictor: cognitive abilities. On a side note, we think this idea can be carried over to other forms of assessment, too. Although it is most easily conducted, and therefore presumably most frequent, with self-report questionnaire methods, faking also occurs with interviews (e.g., phone interviews on drug use, c.f. Colón et al., 2001) or performance measures (faking bad). As an example, for job interviews, faking was described in the context of ATIC (König et al., 2006; Melchers et al., 2009; Kleinmann et al., 2011). Both, ATIC and faking ability, share the same basic idea and, interestingly, similar relations to other cognitive abilities.

Assuming faking ability is the driving factor of faking performance and knowing that personality questionnaires are typically faked in applicant selection (Viswesvaran and Ones, 1999; Birkeland et al., 2006) it can be hypothesized that the incremental predictive validity of personality assessments is due to individual differences in faking ability and therefore due to cognitive abilities, too. While Schmidt and Hunter’s (1998) meta-analysis reports incremental predictive validity of personality assessment over cognitive abilities, which would challenge our hypothesis, we argue that the studies in this meta-analysis are mostly limited in terms of the assessment of cognitive abilities. Typically, the assessment of cognitive abilities in applicant selection is limited to general mental ability only, such as with the widely applied Wonderlic Personnel Test (Wonderlic, 1992), and other abilities, such as crystallized intelligence, are often ignored. In other words, the variance in (faked) personality questionnaires that incrementally predicts job performance might not have incremental predictive power if other predictors than general mental ability were included in tests of incremental predictive validity.

However, we want to stress that this hypothesis was not tested in our studies, but can be derived based on the results of our studies. We encourage future research to answer this research question; either indirectly by exploring the (incremental) predictive validity of faking ability on job performance, or directly by testing the incremental predictive power of personality over a plethora of cognitive abilities that goes beyond the assessment of general mental abilities only. Finally, even if this hypothesis was supported, it does not speak against the general use of personality questionnaires in personnel selection. Even if they do not measure personality in this setting, but certain cognitive abilities, they might be an economic indirect assessment of abilities that are relevant in predicting job performance.

## Ethics Statement

The study was conducted according to the ethical guidelines for online studies of the German Society for Online Research (DGOF, 2007). Consent of each participant was requested in digital form on the first page of the survey and anonymity of participants was guaranteed. Ethical approval was not required as per local legislation.

## Author Contributions

MG contributed to the introduction, task development, study design, scoring, data analysis, and discussion. SO contributed to the introduction, task development, study design, data collection, and discussion. RS contributed to the introduction, task development, study design, and data collection. OW supervised the study and manuscript with supervising contributions to all parts.

## Conflict of Interest Statement

The authors declare that the research was conducted in the absence of any commercial or financial relationships that could be construed as a potential conflict of interest. The reviewer MZ declared a shared affiliation, with no collaboration, with one of the authors, OW, to the handling Editor at time of review.

## Footnotes

1 Doublethink is a neologism presented in the book *Nineteen Eighty-Four* (Orwell, 1949). It refers to pronouncing contradictory ideas or concepts as equally true.

2 https://www.onetonline.org/

3 osf.io/w7qh4

4 https://www.unipark.com/

5 https://www.onetonline.org/
