# Supplementary material for: The “g” in Faking: Doublethink the Validity of Personality Self-Report Measures for Applicant Selection
Source: Front Psychol. 2018 Nov 13;9:2153. doi: 10.3389/fpsyg.2018.02153 (PMC6244170; doi:10.3389/fpsyg.2018.02153)
Supplement: Supplementary file 1 [file Data_Sheet_1.zip › Task Design/Faking Ability Tasks Instruction Examples.pdf]

Appendix B

Instruction B1: *Instruction page before the faking WSQ task – faking pilot. First, a short background story is presented, followed by two attention check questions to assure that participants know what is demanded of them.*

Faking Personality: Commercial Airplane Pilot

Imagine that, after several difficult years of school and training, you are finally finished and have now attained your Commercial Airplane Pilot Certificate.
For your first job, you decide to apply to *American Airlines* to be one of their **commercial pilots**. During the application process, American Airlines tests not only your knowledge and skills, but they are also very interested in your personality. Based on your responses to a personality questionnaire, the personnel staff is trying to identify if you have a personality that best matches those personality traits that are most desired in a commercial airplane pilot.

In this part of the study, we ask you to try to picture the ideal personality of an airplane pilot. What are some distinct personality traits that an airplane pilot should have?

On the next few pages is a personality questionnaire. Please complete the questionnaire with the goal of answering the items so that you convey the ideal personality for a commercial airplane pilot. We want you to try to make the best impression possible and convince *American Airlines* that you are the best candidate, in terms of your personality, for this job. Your responses do not need to reflect your actual personality. You need not be honest about yourself.

The following questionnaire consists of 16 questions. These questions are about work styles. A Work Style is a personal characteristic that can affect how well someone does a job. First, each work style is named and defined.

For example: Stress Tolerance - Job requires accepting criticism and dealing calmly and effectively with high-stress situations.

Then you are asked how important this characteristic is for the performance of the job. To respond, click on a number between 1.00 and 5.00, depending on how important you think the work style is for the performance in the job.

(1) Not important - (2) Somewhat important - (3) Important - (4) Very important - (5) Extremely important.

Please feel free to use the entire scale, including points between the terms.

Before the task begins, please respond to the questions below to assure you have read the instructions carefully.

Are you meant to rate how important these personality traits are for the job of a pilot?

○ yes ○ no

Which job are you pretending to apply for?

○ Dog hairdresser ○ Airplane Pilot

Instruction B2: *Instruction page before the faking NEO subscales task – faking TV announcer. First, a short background story is presented, followed by two attention check questions to assure that participants know what is demanded of them.*

Job Profile Television Announcer:

Imagine you want to be a daytime talk show host. For the past few years, you have gained some experience working for a local television station, reporting the news, weather, the results of local sporting events, and you've even hosted a few charity fundraisers that were broadcasted live on television. Recently, a major network announced they were hiring for a **daytime talk show host**. You believe you have gained enough experience to successfully do that job so you decide to apply. For part of the application process, you are asked to complete a personality questionnaire. The network uses your responses to that questionnaire to decide if you have the ideal personality to be a day time talk show host.

In this part of the study, we ask you to try to picture the ideal personality of a daytime talk show host. What are some distinct personality traits that a daytime talk show host should have?

On the next few pages is a personality questionnaire. Please complete the questionnaire with the goal of answering the items so that you convey the ideal personality for a daytime talk show host. We want you to try to make the best impression possible and convince the network that you are the best candidate, in terms of your personality, for this job. Your responses do not need to reflect your actual personality. You need not be honest about yourself.

The questionnaire consists of of 32 statements used to assess personality attributes, please respond as a television announcer would.

Select "Strongly disagree" if the statement **is definitely false or if you strongly disagree.**

Select "Disagree" if the statement **is mostly false or if you disagree.**

Select "Neutral" if the statement **is about equally true or false**, if you cannot decide, or if you are neutral on the statement.

Select "Agree' if the statement **is mostly true or if you agree**.

Select "Strongly agree" if the statement **is definitely true or if you strongly agree.**

Please feel free to use the entire scale, including points between the terms.

Please respond to the questions below, to assure you have read the instructions carefully.

What are you pretending to be?

- ○ Someone who is eager to make the best impression and get the job
- ○ Someone who only applied for the fun of it

What job are you applying for?

- ○ Professor
- ○ Television Announcer

Instruction B3: *Instruction page before the faking NEO subscales task – faking Tour Guide. First, a short background story is presented, followed by two attention check questions to assure that participants know what is demanded of them.*

Job Profile Tour Guide

Imagine you are an assistant in the local tourism office in your city. For the past year, you have been helping out organizing trips and tours for tourists around the city and the countryside. But now, rather than organizing those trips from the office, you would like to be the actual tour guide and you would like to teach tourists about the history of the town where you live in. You have some experience holding presentations and you take great personal interest in the historic buildings and religious sites of your city. You have decided to apply to your local tourism office for the job of a **tour guide**. As part of the application process, you are asked to complete a personality questionnaire. The tourism office will use your responses on that questionnaire to decide if you have the ideal personality to be a tour guide.

In this part of the study, we ask you to try to picture the ideal personality of a tour guide. What are some distinct personality traits that a tour guide should have?

On the next few pages is a personality questionnaire. Please complete the questionnaire with the goal of answering the items so that you convey the ideal personality for a tour guide. We want you to try to make the best impression possible and convince the tourism office that you are the best candidate, in terms of your personality, for this job. Your responses do not need to reflect your actual personality. You need not be honest about yourself.

The following questionnaire consists of 32 statements, please respond as a tour guide would.

Select "Strongly disagree" if the statement **is definitely false or if you strongly disagree.**

Select "Disagree" if the statement **is mostly false or if you disagree.**

Select "Neutral" if the statement **is about equally true or false**, if you cannot decide, or if you are neutral on the statement.

Select "Agree' if the statement **is mostly true or if you agree.**

Select "Strongly agree" if the statement **is definitely true or if you strongly agree.**

Please feel free to use the entire scale, including points between the terms.

Below are two questions, please answer the questions to make sure you have read the instructions carefully.

What job are you pretending to apply for?

- ◯ A superhero
- ◯ A tour guide

Who are you trying to convince by responding according to the personality attributes most important for a tour guide?

- ◯ You are trying to convince the employers at the coffee shop
- ◯ You are trying to convince the employers at the museum
