# Supplementary material for: The “g” in Faking: Doublethink the Validity of Personality Self-Report Measures for Applicant Selection
Source: Front Psychol. 2018 Nov 13;9:2153. doi: 10.3389/fpsyg.2018.02153 (PMC6244170; doi:10.3389/fpsyg.2018.02153)
Supplement: Supplementary file 1 [file Data_Sheet_1.zip › Task Design/Excluded Measures - Job Knowledge Task Example Item.docx]

**Faking Task 1 – Job Knowledge Test**

This task asks participants to choose most or least important work styles for a certain job among three options in multiple choice items. An example item reads:

- Which of these three personality attributes is most important for the job of an airplane pilot?
- A) Analytical Thinking B) Social Orientation C) Innovation

Here, the correct answer would be A), because Analytical Thinking has, among those three, the highest mean expert score (AM=4.52) in the O*NET database. Items were designed for all three jobs, always based on Work Styles from the WSQ. To ensure adequate distance between the correct answer and the distractors, they were chosen in a way, that their 90%-CIs of expert ratings did not overlap.

Each task consisted of six items.
